# The eIF2α serine 51 phosphorylation-ATF4 arm promotes HIPPO signaling and cell death under oxidative stress

**DOI:** 10.18632/oncotarget.10480

**Published:** 2016-07-07

**Authors:** Kamindla Rajesh, Jothilatha Krishnamoorthy, Jyotsana Gupta, Urszula Kazimierczak, Andreas I. Papadakis, Zhilin Deng, Shuo Wang, Shinji Kuninaka, Antonis E. Koromilas

**Affiliations:** ^1^ Lady Davis Institute for Medical Research, McGill University, Sir Mortimer B. Davis-Jewish General Hospital, Montreal, Quebec, Canada; ^2^ Department of Cancer Immunology, Chair of Medical Biotechnology, Poznan University of Medical Sciences, Poznan, Poland; ^3^ Division of Gene Regulation, Institute for Advanced Medical Research, Keio University School of Medicine, Tokyo, Japan; ^4^ Department of Oncology, Faculty of Medicine, McGill University, Montreal, Quebec, Canada

**Keywords:** translation initiation factor eIF2, protein phosphorylation, activating transcription factor 4, mRNA translation, large tumor suppressor 1

## Abstract

The HIPPO pathway is an evolutionary conserved regulator of organ size that controls both cell proliferation and death. This pathway has an important role in mediating cell death in response to oxidative stress through the inactivation of Yes-associated protein (YAP) and inhibition of anti-oxidant gene expression. Cells exposed to oxidative stress induce the phosphorylation of the alpha (α) subunit of the translation initiation factor eIF2 at serine 51 (eIF2αP), a modification that leads to the general inhibition of mRNA translation initiation. Under these conditions, increased eIF2αP facilitates the mRNA translation of activating transcription factor 4 (ATF4), which mediates either cell survival and adaptation or cell death under conditions of severe stress. Herein, we demonstrate a functional connection between the HIPPO and eIF2αP-ATF4 pathways under oxidative stress. We demonstrate that ATF4 promotes the stabilization of the large tumor suppressor 1 (LATS1), which inactivates YAP by phosphorylation. ATF4 inhibits the expression of *NEDD4.2* and *WWP1* mRNAs under pro-oxidant conditions, which encode ubiquitin ligases mediating the proteasomal degradation of LATS1. Increased LATS1 stability is required for the induction of cell death under oxidative stress. Our data reveal a previously unidentified ATF4-dependent pathway in the induction of cell death under oxidative stress via the activation of LATS1 and HIPPO pathway.

## INTRODUCTION

The HIPPO pathway is conserved from *Drosophila* to mammals and is involved in cell growth, proliferation, apoptosis, organ size and tumorigenesis [1]. It consists of two groups of kinases, the mammalian STE20-like protein kinase 1 (MST1) and MST2, and the large tumor suppressor 1 (LATS1) and LATS2, in combination with their activating adaptor proteins, Salvador family WW domain-containing protein 1 (SAV1) and MOB kinase activator 1 (MOB1), respectively [1]. The transcriptional module of the pathway is composed of the transcriptional co-activators yes-associated protein (YAP) and its paralogue, transcriptional co-activator with PDZ-binding motif (TAZ), and TEA domain family members (TEAD1-4). When the upstream kinase module is activated, LATS1 and LATS2 phosphorylate YAP/TAZ at multiple sites [2], which leads to inhibition of transcriptional activity through 14-3-3-mediated cytoplasmic retention of YAP/TAZ and its proteasomal degradation [3]. When YAP/TAZ is not phosphorylated by the LATS kinases, they translocate to the nucleus and bind to sequence specific transcription factors TEAD1-4 (and other transcription factors including SMAD, RUNX, TP73, FOXO1), which enables the transcription of genes involved in proliferation and survival [3].

Regulation of mRNA translation is one of the most immediate cell responses to any form of stress [4]. Cells respond to various stress forms by blocking the initiation process *via* the phosphorylation of eIF2α at serine (S) 51 (herein referred to as eIF2αP), a modification that leads to global inhibition of protein synthesis [4, 5]. Induction of eIF2αP is mediated by a family of four kinases each of which is activated by different forms of stress and is part of a biological process known as the integrated stress response (ISR) [5, 6]. The family consists of the heme-regulated inhibitor (HRI) activated by heme-deficiency in erythrocytes; the protein kinase RNA-dependent kinase (PKR) activated by double stranded (ds) RNA and virus infection; the PKR-like endoplasmic reticulum (ER) resident kinase (PERK) activated by the accumulation of misfolded proteins in the ER; and the general control non-derepressible 2 (GCN2) activated by uncharged tRNAs from amino acid deprivation [5, 6]. Increased eIF2αP leads to a global inhibition of mRNA translation but also facilitates translation of select mRNAs synthesizing proteins with key roles in adaptation to stress [7]. That is, mRNAs encoding for the activating transcription factor 4 (ATF4) and ATF5 in mammalian cells, or general control non-repressed 4 (GCN4) in yeast, are better translated under conditions of increased eIF2αP through delayed translation re-initiation from upstream open reading frames (uORFs) within the 5′ untranslated region (5′ UTR) [8–10].

Oxidative stress occurs when the equilibrium between cellular production of pro-oxidants and anti-oxidant defense mechanisms is disrupted leading to accumulation of reactive oxygen species (ROS), including the superoxide radical O_2_^.−^, hydrogen peroxide H_2_O_2_, and the highly reactive hydroxyl radical ^.^OH [11]. Cells cope with ROS by increasing the expression of anti-oxidant genes and activating pathways that control survival and adaptation to oxidative stress [11]. The HIPPO pathway has been implicated in the induction of cell death under oxidative stress. Oxidative stress activates MST1 by disrupting its interaction with Thioredoxin-1 or by promoting its phosphorylation by c-ABL leading to the phosphorylation of the forkhead transcription factor FOXO3 and increased expression of the pro-apoptotic gene BIM in neuronal cells [12–14]. ROS production by ischaemia/reperfusion results in cardiomyocyte death *via* the activation of MST1 and inactivation of the YAP and FOXO3 transcriptional complex, which limits the expression of anti-oxidant genes and promotes oxidative stress-mediated cell death [15–17]. On the other hand, induction of eIF2αP plays a pivotal role in the regulation of redox homeostasis and adaptation of eukaryotic cells to oxidative stress [18–22]. eIF2αP decreases ROS production, prevents the appearance of premature senescence in primary fibroblasts and protects tumor cells from death caused by pro-oxidant drugs [19]. ATF4, which is efficiently translated under conditions of enhanced eIF2αP, mediates the transcriptional induction of several genes encoding for proteins controlling anti-oxidant responses, amino acid import and protein synthesis, which contribute to either the adaptation of cells to oxidative stress or induction of death under excessive oxidative stress [21, 23]. Herein, we demonstrate that the eIF2αP-ATF4 arm activates the HIPPO pathway to promote death in response to oxidative stress. This is mediated by the ability of ATF4 to suppress the expression of E3 ubiquitin ligases that mediate the destabilization of LATS1 leading to increased HIPPO signaling and induction of oxidative cell death.

## RESULTS

### Increased eIF2αP facilitates LATS1 expression under oxidative stress

**Figure 1:**
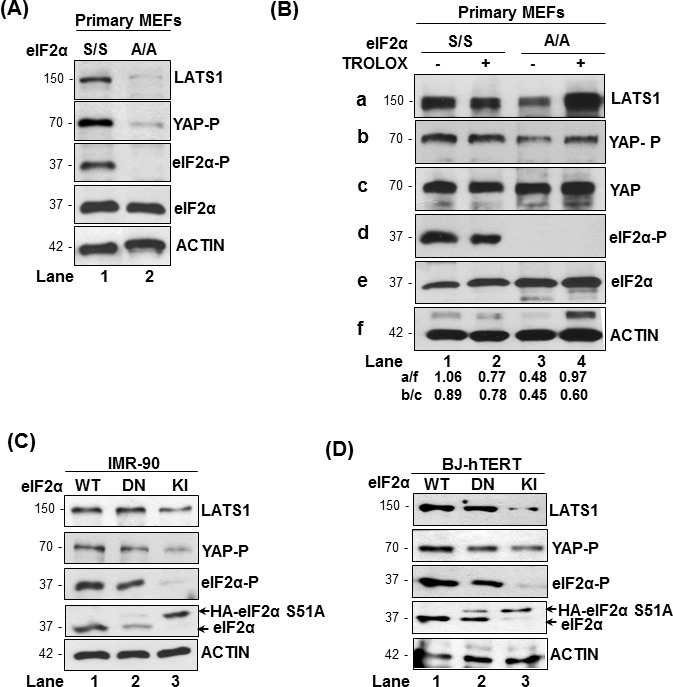
eIF2αP promotes LATS1 expression in mouse and human fibroblasts (A, B) Primary MEFs containing either wild type (S/S) or the S51A mutant eIF2α allele (A/A) were left untreated **A.** or treated with 200 μM Trolox **B.**. **C.**, **D.** Human IMR-90 **C.** and BJ-hTERT fibroblasts **D.** containing endogenous eIF2α (wild type; WT; lane 1) or the non-phosphorylated dominant negative (DN) HA-eIF2α S51A in the absence (lane 2) or presence of shRNA targeting the 3′ untranslated region (3′ UTR) of endogenous eIF2α (knock-in; KI; lane 3). **A.**-**D.** Protein extracts (50 μg) were subjected to immunoblot analyses for the indicated proteins. The approximate molecular size of the proteins as well as the position of HA-eIF2α S51A in the polyacrylamide gels compared to endogenous eIF2α is indicated. eIF2α-P, S51 phosphorylation; YAP-P, S127 phosphorylation.

We examined LATS1 expression and YAP phosphorylation in primary mouse embryonic fibroblasts as well as primary and immortalized human fibroblasts that were genetically engineered to be impaired in eIF2αP [19]. We observed that LATS1 protein was downregulated in primary mouse embryonic fibroblasts (MEFs) bearing a homozygous S51A allele of eIF2α (eIF2αA/A) compared to primary MEFs with wild type eIF2α allele (eIF2αS/S; Figure 1A) [19]. Decreased LATS1 was associated with decreased phosphorylation of YAP at S127, which is mediated by LATS1 and implicated in the inactivation of YAP [24]. Based on our previous study demonstrating that impaired eIF2αP in primary MEFs leads to increased ROS production [19], we examined whether ROS was responsible for LATS1 downregulation in eIF2αA/A MEFs. Incubation of primary eIF2αS/S and eIF2αA/A MEFs with Trolox, which is a derivative of vitamin E with anti-oxidant function [25], prevented the downregulation of LATS1 and inhibition of YAP S127 phosphorylation in eIF2αA/A MEFs compared to eIF2αS/S MEFs (Figure 1B). These data suggested that loss of eIF2αP decreases the expression of LATS1 as a result of increased oxidative stress.

We also employed human IMR-90 and TERT)-immortalized BJ (BJ-hTERT) fibroblasts, which were shown by our group to contain increased ROS under conditions of impaired eIF2αP [19]. The human fibroblasts were engineered to express an HA-tagged form of the non-phosphorylatable eIF2αS51A mutant with dominant negative (DN) function (Figure 1C, 1D) [19]. The DN-expressing fibroblasts were further modified to be deficient in eIF2αP by the expression of a short-hairpin (sh) RNA against the 3′ untranslated region of the endogenous eIF2α mRNA [19]. This approach generated human fibroblasts predominantly expressing the non-phosphorylated HA-eIF2αS51A mutant, which are referred to as knock-in (KI) cells (Figure 1C, 1D). We noticed that IMR-90 and BJ-hTERT KI fibroblasts exhibited decreased levels of LATS1 and YAP S127 phosphorylation compared to fibroblasts with intact eIF2α (Figure 1C, 1D). Considering that inhibition of eIF2αP results in increased ROS in the human fibroblasts [19], the data indicated that LATS1 downregulation in eIF2αP-deficient cells depends, at least in part, on ROS.

**Figure 2:**
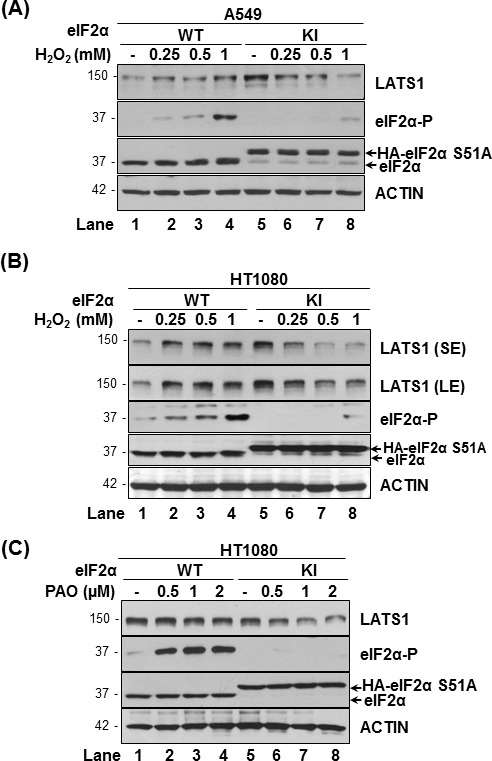
eIF2αP facilitates LATS1 expression in tumor cells under oxidative stress A549 cells **A.** and HT1080 cells **B.**, **C.** that were either proficient (wild type; WT) or deficient for eIF2αP(knock-in; KI) were exposed to the indicated concentrations of H_2_O_2_ for 2 h **A.**-**B.** as well as the pro-oxidant drug PAO for 15 min **C.**. Protein extracts (50 μg) were immunoblotted for the indicated proteins. The approximate molecular size of the proteins as well as the position of HA-eIF2α S51A and endogenous eIF2α in the polyacrylamide gels is indicated.

We previously demonstrated that tumor cells with impaired eIF2αP are adapted to increased intracellular ROS but become increasingly sensitive to the anti-proliferative effects of extrinsic ROS [18, 19]. We observed that human lung tumor A549 cells and human fibrosarcoma HT1080 cells displayed increased eIF2αP and LATS1 in response to increasing concentrations of hydrogen peroxide (H_2_O_2_) (Figure 2A, 2B). On the other hand, LATS1 expression was downregulated by the H_2_O_2_ treatments in eIF2αP-deficient A549 and HT1080 cells indicating that eIF2αP is required for LATS1 upregulation under oxidative stress. The ability of eIF2αP to promote LATS1 expression became further evident in experiments with the pro-oxidant drug phenylarsine oxide (PAO), which decreased LATS1 in the eIF2αP-deficient but not proficient HT1080 cells (Figure 2C) [18, 26].

### ATF4 mediates LATS1 stabilization under oxidative stress

**Figure 3:**
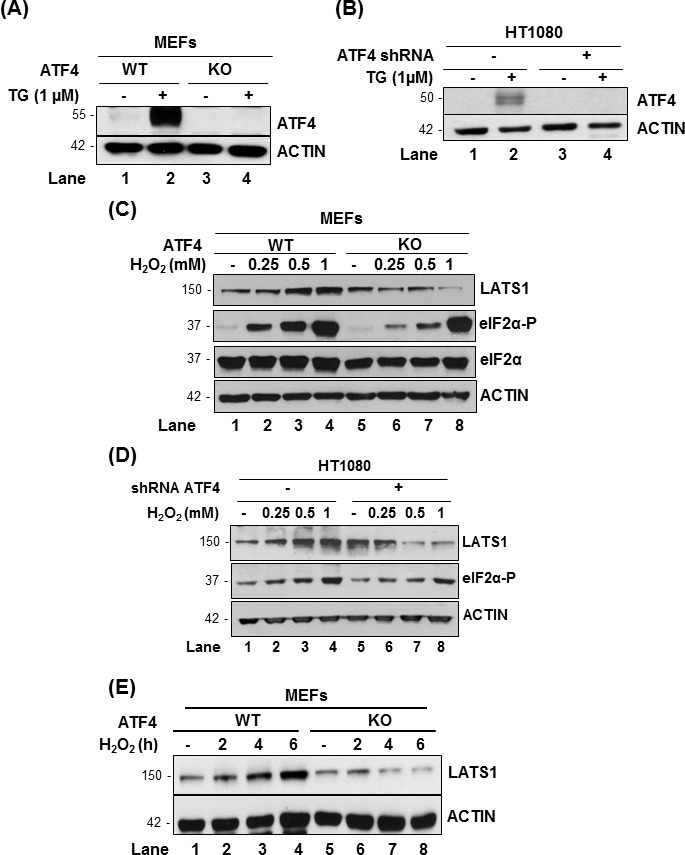
ATF4 promotes LATS1 expression under pro-oxidant conditions **A., B.** Immortalized MEFs containing (WT) or lacking ATF4 (knockout; KO) as well as HT1080 cells lacking (−) or expressing ATF4 shRNA (+) were subjected to 1 μM thapsigargin (TG) for 2h. **C.**, **D.** ATF4 WT and KO MEFs as well as HT1080 cells lacking or expressing ATF4 shRNA were treated with the indicated amounts of H_2_O_2_ for 2h. **E.** ATF4 WT and KO MEFs were treated with 0.5mM H_2_O_2_ for the indicated time points. **A.**-**E.** Protein extracts (50 μg) were immunoblotted for the indicated proteins. The approximate molecular size of the proteins in the polyacrylamide gels is indicated.

ATF4 plays a key role in mediating the biological effects of eIF2αP in response to oxidative stress [21, 27]. We employed immortalized MEFs that were either proficient (WT) or knockout (KO) of ATF4 as well as HT1080 cells, which were engineered to be deficient for ATF4 by shRNA expression [18]. In both cell types, ATF4 deficiency was confirmed by the immunoblotting of ATF4 protein in extracts from cells subjected to ER stress by thapsigargin (Figure 3A, 3B). When cells were exposed to H_2_O_2_, we noticed that LATS1 was upregulated in ATF4-proficient MEFs and HT1080 cells as opposed to ATF4-deficient cells in which LATS1 was downregulated with increasing concentrations of H_2_O_2_ (Figure 3C, 3D). A similar effect of ATF4 on LATS1 expression was observed in MEFs exposed to H_2_O_2_ for different time points (Figure 3E). We further noticed that induction of eIF2αP was proportional to the concentrations of H_2_O_2_ in ATF4-proficient MEFs and HT1080 cells (Figure 3C, 3D). However, ATF4-deficiency resulted in decreased eIF2αP in the mouse and human cells after H_2_O_2_ treatment compared to the isogenic control cells through as yet unclear mechanism (Figure 3C, 3D).

**Figure 4:**
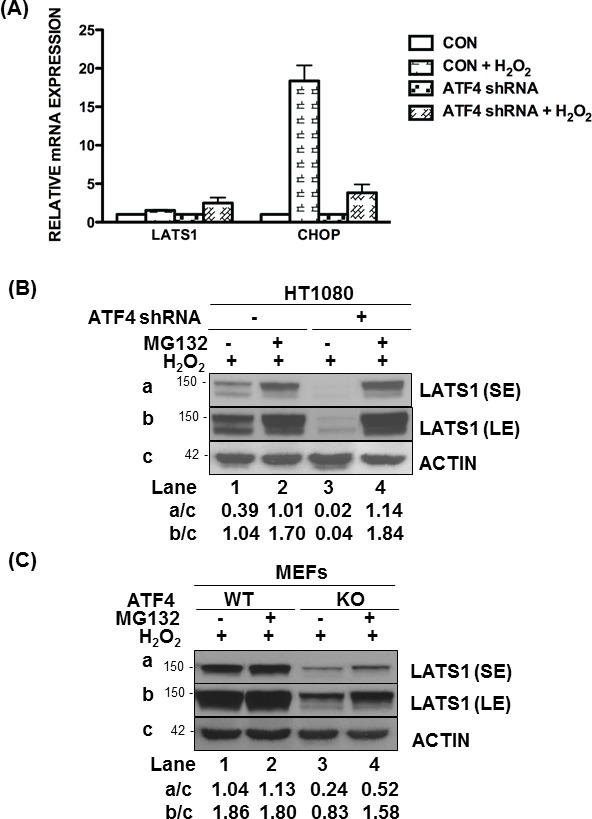
ATF4 mediates LATS1 stabilization under oxidative stress **A.** Detection of *LATS1* and *CHOP* mRNAs by qPCR in ATF4-proficient and deficient (shRNA expressing) HT1080 cells exposed to 0.5mM H_2_O_2_ for 8h. **B.**, **C.** ATF4-proficient or deficient (shRNA expressing) HT1080 cells as well as ATF4 WT or KO MEFs were untreated or pre-treated with 25 μM of MG132 for 1h followed by treatment with 0.25mM H_2_O_2_ in the absence or presence of MG132 for 4h. **B.**, **C.** Protein extracts (50 μg) were immunoblotted for the indicated proteins. The approximate molecular size of the proteins in the polyacrylamide gels is indicated. The ratios of quantified bands for each lane are indicated. SE, short blot exposure; LE, long blot exposure.

We further examined whether *LATS1* was under the transcriptional control of ATF4. *LATS1* mRNA levels did not significantly differ between ATF4-proficient and deficient HT1080 cells subjected to H_2_O_2_ treatment (Figure 4A). Under these conditions, induction of the DNA damage-inducible transcript 3, also known as C/EBP homologous protein (*CHOP*), which is an ATF4-dependent gene in stressed cells [23], was impaired in ATF4-deficient compared to proficient HT1080 cells treated with H_2_O_2_ (Figure 4A). Thus, ATF4 is unlikely to play a role in LATS1 expression at the transcriptional level. Because LATS1 expression is tightly controlled by post-translational modifications [28], we sought to examine the role of ATF4 in this process. Treatment with the proteasome inhibitor MG132 resulted in a higher induction of LATS1 expression in ATF4-deficient than proficient HT1080 cells and MEFs in response to H_2_O_2_ treatment (Figure 4B, 4C). This finding implicated ATF4 in the stabilization of LATS1 protein under oxidative stress.

### ATF4 inhibits NEDD4.2 and WWP1 expression under oxidative stress

**Figure 5:**
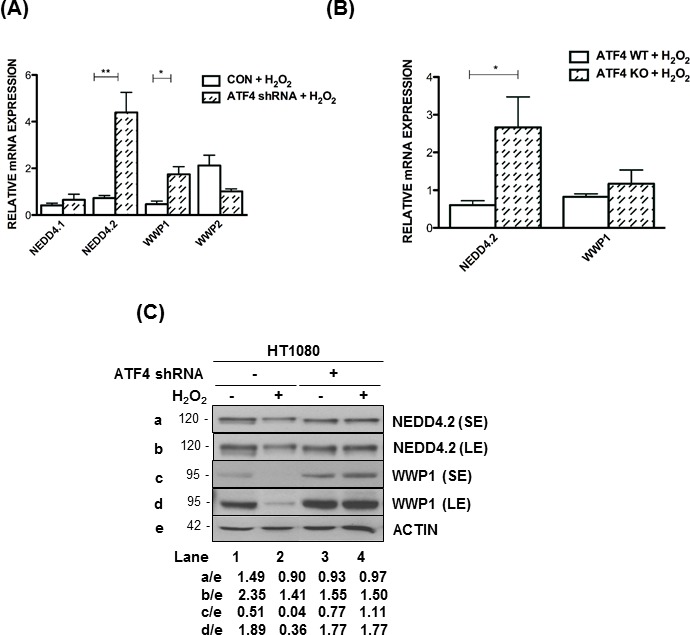
ATF4 decreases NEDD4 and WWP1 expression under oxidative stress **A.**, **B.** Quantification of mRNA levels by qPCR of the indicated E3 ubiquitin ligase genes in HT1080 cells **A.** or MEFs **B.** subjected to H_2_O_2_ treatment after normalization to mRNA levels in untreated cells. Values represent arithmetical mean ± SE for individual gene from 3 independent experiments. *P < 0.05; **P < 0.01 **C.** ATF4-proficient and ATF4 shRNA-expressing HT1080 cells were left untreated or treated with 0.5mM H_2_O_2_ for 4h. Protein extracts (50 μg) were immunoblotted for the indicated proteins. The approximate molecular size of the proteins in the polyacrylamide gels is indicated. The ratios of protein intensities for each lane are indicated. SE, short exposure of the blot; LE, long exposure of the blot.

Several studies established that members of the neural precursor cell expressed developmentally down-regulated 4 (NEDD4) family of the E3 ubiquitin-protein ligases play key roles in LATS1 destabilization [29, 30]. We observed that among the different NEED4 family members *NEDD4.2* and WW domain containing E3 ligase 1 (*WWP1*) mRNA levels were substantially increased in ATF4-deficient compared to proficient HT1080 cells in response to H_2_O_2_ treatment (Figure 5A). We also observed that *NEED4.1* and *WWP2* mRNA levels were not induced in ATF4-deficient HT1080 cells under oxidative stress (Figure 5A). Moreover, *NEDD4.2* mRNA levels were upregulated in ATF4 KO MEFs as opposed to *WWP1* mRNA levels, which were marginally increased by the loss of ATF4 (Figure 5B). Immunoblot analyses showed the downregulation of NEDD4.2 and WWP1 in ATF4-proficient but not deficient HT1080 cells in response to H_2_O_2_ treatment (Figure 5C). It was of interest that ATF4 mediated a stronger inhibitory effect on the protein than the mRNA levels of WWP1 suggesting that ATF4 may also inhibit WWP1 expression at the translational and/or post-translational level. These data revealed the ability of ATF4 to inhibit the expression of E3 ubiquitin ligases involved in LATS1 degradation under oxidative stress.

### LATS1 promotes the induction of cell death under oxidative stress

**Figure 6:**
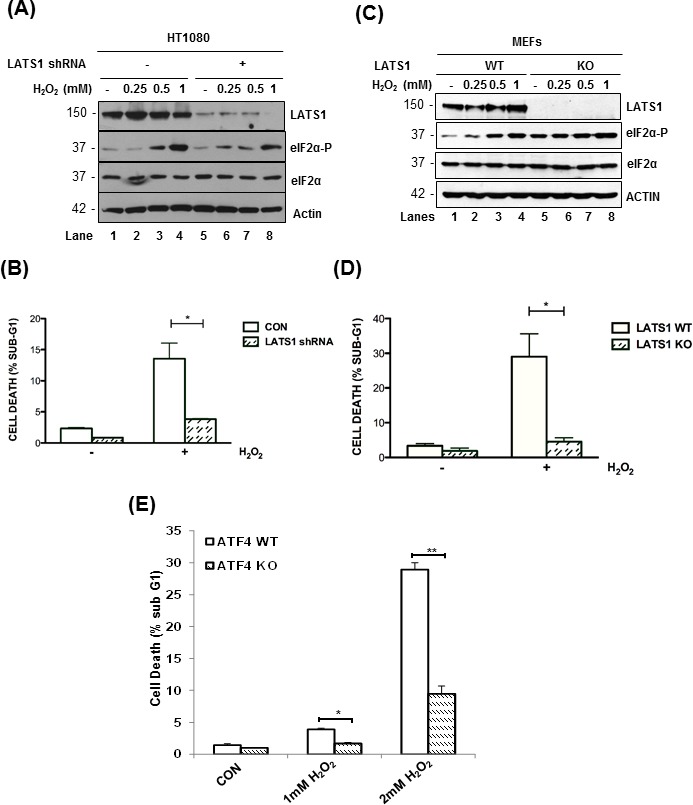
LATS1 promotes cell death in response to oxidative stress LATS1-proficient and LATS1 shRNA-expressing HT1080 cells **A.** or LATS1 KO MEFs **B.** were subjected to treatments with the indicated concentrations of H_2_O_2_ for 2 h. **A.**, **C.** Protein extracts (50 μg) were immunoblotted for the indicated proteins. The approximate molecular size of the proteins in the polyacrylamide gels is indicated. **B.**, **D.** LATS1-proficient and deficient HT1080 cells **B.** as well as LATS1 WT and KO MEFs **D.** were exposed to 1 mM H_2_O_2_ for 8 h. **E.** ATF4 WT and KO MEFs were subjected to treatments with the indicated concentrations of H_2_O_2_ for 8 h. **B.**-**E.** Cell death was assessed by the percentage of cells in the sub-G_1_ population by propidium iodide staining and FACS analysis. Values represent the arithmetic mean ± SE from 3 independent experiments. *P < 0.05; **P < 0.01.

Recent studies showed that induction of the HIPPO pathway and YAP inactivation by oxidative stress promotes human cardiomyocyte death through LATS2 activation [15, 16]. Similarly, we found that LATS1 downregulation by shRNA substantially decreased the susceptibility of HT1080 cells to death by H_2_O_2_ (Figure 6A, 6B). Also, immortalized MEFs from LATS1 KO mice exhibited an increased resistance to H_2_O_2_-mediated death compared to their isogenic LATS1 WT counterparts (Figure 6C, 6D). We observed that LATS1 inactivation in HT1080 cells mitigated the induction of eIF2αP in response to H_2_O_2_-treatment (Figure 6A). On the other hand, LATS1 depletion in MEFs increased the basal levels of eIF2αP but decreased its further induction upon oxidative stress compared to LATS1-proficient MEFs (Figure 6C). The effect of LATS1 inactivation on eIF2αP was similar to that observed with LATS1 downregulation in ATF4-deficient MEFs indicating the presence of a LATS1-orchestrated feedback loop leading to increased eIF2αP and LATS1 stabilization under oxidative stress (Figure 3C, 3D). Furthermore, ATF4 KO MEFs were substantially resistant to death to H_2_O_2_ compared to ATF4 WT MEFs supporting the notion that ATF4 and LATS1 act in the same pathway to induce cell death under oxidative stress (Figure 6E).

## DISCUSSION

Previous studies by our group established that increased eIF2αP by PERK and GCN2 under oxidative stress mediates the induction of pro-survival responses that depend on AKT activation [18, 21]. AKT exhibits pro-survival functions but its activation under conditions of excessive oxidative stress can also lead to induction of either premature senescence or cell death [31], both of which are antagonized by eIF2αP [18, 32]. Thus, eIF2αP promotes the pro-survival and inhibits the pro-death functions of AKT in cells subjected to oxidative stress [19, 32]. The ability of eIF2αP to mediate LATS1 stabilization, which promotes death under oxidative stress, indicates the presence of a pathway that counterbalances the pro-survival effects of eIF2αP in cells exposed to oxidative insults.

**Figure 7:**
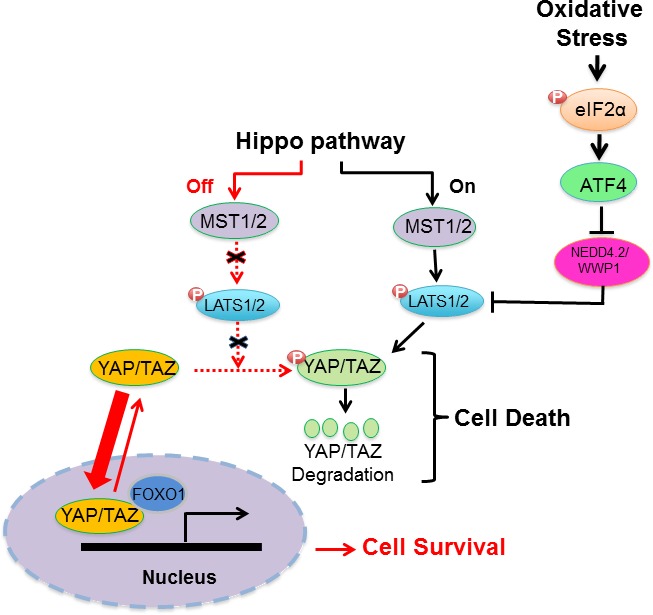
Schematic representation of regulation of HIPPO pathway by the eIF2αP-ATF4 arm under oxidative stress Induction of eIF2αP in cells subjected to oxidative stress leads to ATF4 upregulation at the translational level, which in turn inhibits the expression of E3 ubiquitin ligases NEDD4.2 and WWP1 that target LATS1 for degradation [29, 30]. LATS1 stabilization results in the phosphorylation and cytoplasmic sequestration of YAP/TAZ, which impairs YAP/TAZ-dependent expression of genes involved in cell survival and proliferation. Interaction of YAP/TAZ with FOXO1 has been demonstrated to play a key role in the induction of anti-oxidant genes and cell survival under oxidative stress [16].

ATF4 mediates the expression of genes involved in amino acid import, glutathione biosynthesis, and resistance to oxidative stress [21]. Also, ATF4 hyper-activation has been implicated in the upregulation of protein synthesis, ATP depletion and increased oxidative stress leading to cell death [23, 33]. Herein, we demonstrate that the pro-death effects of ATF4 under oxidative stress are linked to LATS1 stabilization and activation of the HIPPO pathway (Figure 7). In cells subjected to oxidative stress, ATF4 suppresses the expression of the E3 ubiquitin ligases NEDD4.2 and WWP1, both of which target LATS1 for degradation (Figure 5) [29, 30]. LATS1 and other components of the HIPPO pathway are under the control of E3 ubiquitin ligases of the NEDD4 family including NEDD4.1, NEDD4.2, ITCH and WWP1 that can determine the activation of the pathway in response to different environmental cues [29, 30, 34–37]. The WW domains of the E3 ubiquitin ligases mediate an interaction with the PPxY motif of LATS1 [38], which plays a key role in LATS1 degradation and increased YAP transcriptional activity [29, 30, 35]. Our data show that ATF4 inhibits *NEDD4.2* and *WWP1* but not *NEDD4.1* and *WWP2* mRNA levels indicating its role in the inhibition of specific E3 ubiquitin ligases under oxidative stress (Figure 5). ATF4 appears to function as a transcriptional repressor of *NEDD4.2* and WWP1, which is line with its ability to inhibit the expression of several genes in stress-regulated genes including members of the NEDD4 family [23]. Our findings are in line with recent studies demonstrating that HIPPO activation and YAP inactivation by oxidative stress promotes the death of human cardiomyocytes through LATS2 activation [15, 16]. The ability of the HIPPO pathway to cause death under oxidative stress has been attributed to the inactivation of YAP and inhibition of expression of anti-oxidant genes mediated by the interaction of YAP with FOXO1 [16]. Thus, HIPPO pathway activation downstream of ATF4 contributes to cell death and may be an important determinant of the cell fate decisions of ATF4 in cells under oxidative stress (Figure 7).

A recent study indicated the presence of a crosstalk between eIF2αP and HIPPO signaling in cells subjected to ER stress [39]. Specifically, the PERK-eIF2αP-ATF4 arm cooperates with YAP to increase cell survival under transient ER stress [39]. However, prolonged ER stress activates the HIPPO pathway, which promotes the expression of the growth arrest and DNA damage-inducible protein 34 (GADD34) subunit of protein phosphatase 1 (PP1) resulting in eIF2αP de-phosphorylation and increased susceptibility of stressed cells to death [39]. The functional crosstalk between the PERK-eIF2αP-ATF4 and HIPPO pathways under ER stress was found to be implicated in liver size and tumorigenesis [39]. However, the effects elicited by the activation of HIPPO pathway under ER stress may not depend on LATS1/2 inasmuch as other kinases downstream of MST1 and 2 such as the nuclear dbf2-related (NDR) kinase have been implicated in YAP phosphorylation and inhibition of liver and colon cancer [40, 41]. Also, ER stress induces the expression of seven in absentia homolog 2 (SIAH2), a ubiquitin ligase that has been implicated in ATF4 stabilization and induction of cell death [42]. SIAH2 was shown to lead to LATS2 destabilization and YAP activation in hypoxic cells [43], an effect that provides a tentative connection between ATF4 and HIPPO pathway in stressed cells in the tumor microenvironment. Our data show that, unlike ER stress, the eIF2αP-ATF4 arm contributes to HIPPO activation and cell death. Also, different from ER stress, LATS1 stabilization does not decrease but rather increases eIF2αP indicating a positive effect of HIPPO activation on eIF2αP under oxidative stress (Figure 6). Collectively, the findings indicate that the eIF2αP-ATF4 arm mediates distinct effects on the HIPPO pathway in response to different forms of stress.

Previous work demonstrated that increased ROS levels cause the activation of protein kinase C delta (PKCδ) leading to downregulation of LATS1 and impaired cytokinesis [44]. A recent study indicated that inactivation of PKR causes a defect in cytokinesis raising the intriguing hypothesis that such an effect of the eIF2α kinase could be mediated, at least in part, by LATS1 downregulation [45]. Blockade of cytokinesis is thought to act as barrier to cellular immortalization ensuring stable cell-cycle arrest in senescent cells [45], an effect that could account for the inability of eIF2αP- and ATF4-deficient cells to bypass senescence and undergo oncogenic transformation [19, 46].

There has been growing and strong evidence to suggest that HIPPO is a tumor suppressor pathway. LATS1 KO mice develop fibrosarcomas by 4-10 months of age whereas disruption of other key components of the HIPPO pathway has led to the development of many tumor forms in mice including hepatocellular carcinoma, Schwannoma, squamous cell carcinoma or malignant mesothelioma [47, 48]. On the other hand, YAP/TAZ exhibits oncogenic properties and its hyper-activation is observed in many forms of human cancers [48]. Studies aimed at targeting the HIPPO pathway in cancer are still in infancy; pharmacological inhibition of YAP/TAZ-TEAD complex formation by drugs like verteporfin is thought to be an efficient way to activate HIPPO signaling and is an emerging anti-tumor treatment with promising results in tissue culture assays and mouse models of cancer [49, 50]. On the other hand, eIF2αP is intimately involved in the survival and adaptation of cells exposed to oncogenic insults and promotes tumor survival in response to chemotherapeutic drugs or radiotherapy [6, 51]. The functional interplay between the eIF2αP-ATF4 and HIPPO pathways under oxidative stress may provide a potential nodal point for anti-tumor intervention with pro-oxidant therapies. For example, pro-oxidant therapies may simultaneously induce the pro-survival effects of eIF2αP through the activation of AKT and pro-death effects of ATF4 *via* LATS1 stabilization and HIPPO activation. Pharmacological manipulation of the two opposing pathways may improve anti-tumor response to pro-oxidant therapies and prove an effective means to treat cancers with high content of ROS.

## MATERIALS AND METHODS

### Cell culture and treatments

Primary as well as immortalized eIF2αP-proficient or deficient MEFs, IMR90, BJ-hTERT, HT1080, and A549 cells were generated and maintained as described previously [18, 19]. ATF4 KO MEFs as well as HT1080 cells expressing ATF4 shATF4 were established as previously described [18]. LATS1 KO MEFs were previously reported [52]. The shRNA-mediated knockdown of LATS1 in HT1080 cells was performed with as previously described targeting vector [53]. Cells were cultured in Dulbecco modified Eagle medium (DMEM; Wisent) supplemented with 10% fetal bovine serum (FBS; Gibco), antibiotics (100 U/ml of penicillin-streptomycin; Gibco) and 2.5 μg/ml of puromycin (Sigma). H_2_O_2_ was purchased from Bioshop, Canada; thapsigargin, trolox, phenylarsine oxide (PAO) and propidium iodide were obtained from Sigma; MG132 was purchased from Enzo Life Sciences. In control treatments, an equivalent volume of the solvent of each drug was added in culture.

### Flow cytometry analysis

Cells were subjected to propidium iodide staining and FACScan analysis based on a previously described protocol [18]. FACS was performed with BD FACScalibur and the data was analyzed using the FlowJo software (Tree Star inc).

### Western blot analysis

Protein extraction, quantification and immunoblotting were performed as previously described [18, 19]. The primary antibodies rabbit monoclonal against phosphorylated eIF2α at S51 (Novus Biologicals), mouse monoclonal against eIF2α (Cell Signaling Tech.) rabbit polyclonal against YAP phosphorylated at S127 (Cell Signaling Tech.), rabbit monoclonal against YAP (Cell Signaling), rabbit monoclonal against LATS1 (Cell Signaling Tech.), mouse monoclonal antibody against actin (Clone C4, ICN Biomedicals Inc) rabbit monoclonal against NEDD4.2 (Abcam), rabbit polyclonal against WWP1 or ATF4 (Proteintech) were used. All antibodies were used at a final concentration of 0.1-1 μg/ml. Following incubation with the indicated primary antibodies, membranes were probed with either anti-mouse or anti-rabbit IgG antibodies conjugated to horseradish peroxidise (HRP) (Mandel Scientific). Proteins were visualized with the enhanced chemiluminescence (ECL) reagent (Thermo Scientific) detection system according to the manufacturer's instructions. Quantification of bands in linear range of exposure was performed by densitometry using Scion image software (Frederick, Maryland, USA).

### RNA extraction, reverse transcriptase assay and real-time PCR

RNA was extracted using TRIzol reagent (Invitrogen) according to manufacturer's instructions. This was followed by cDNA preparation using the superscript III RNase H-reverse transcriptase (Invitrogen) as provided in the manufacturer's manual. Real-time PCR was performed on a ThermoCycler instrument (Eppendorf) using the SensiFast SYBR Green kit (FroggaBio). The following oligonucleotides were used for the amplification:

Human ACTIN (Forward 5′-CAGCAGATGTGGATCAGCAAG-3′; Reverse 5′-GCATTTGCGGTGGACGAT-3′); Human LATS1 (Forward 5′-GGCTTCAGATGGACACACGAT-3′ ; Reverse 5′-CCACATCGACAGCTTGAGGG-3′); Human NEDD4.1 (Forward 5′-CACATCTCGGGTGCCTATGA-3′; Reverse 5′-TCAGGAGTACCCCACTGTTCA-3′); Human NEDD4.2 (Forward 5′-CAATGGGGCAGTCCTGAGAA-3′; Reverse 5′-TTCCACGGCCATGAGAAGTT-3′); Human WWP1 (Forward 5′-TTGCTGAGCTCATGGGAAGT-3′; Reverse 5′-TGGTGGTAGATCCAAGCGAT-3′); Human WWP2 (Forward 5′- CGTCAAGAACTCAGGCCACA-3′; Reverse 3′- CAACGGAAGGTTCTTCGGGA-3′); Human CHOP (Forward 5′-GAGCTGGAACCTGAGGAGAGA-3′; Reverse 5′-TGCAGTTGGATCAGTCTGCTT-3′); Mouse NEDD4.1 (Forward 5′-GGGGCAGTCCCGAAAAACTA-3′; Reverse 5′-GGATTGTGGTCCATTCGAGC-3′); Mouse NEDD4.2 (Forward 5′-GGGGCAGTCCCGAAAAACTA-3′; Reverse 5′-CTTGAGCGTTTTCCACAGCC-3′); Mouse WWP1 (Forward 5′-TTGCTGAGCTCATGGGAAGT-3′; Reverse 5′-TGGTGGTAGATCCAAGCGAT-3′); Mouse CHOP (Forward 5′-AGTTATCTTGAGCCTAACACGTC-3′; Reverse 5′-CACTTCCTTCTGGAACACTCTCTC-3′)

### Statistical analysis

Statistical analysis was performed using Graph Pad Prism 5.0. Error bars represent standard error (SE). Significance in differences between arrays of data tested was determined using two-tailed Student ‘t’ test.
